# Outcomes of Bariatric Surgery in Hypothyroid Patients Taking Levothyroxine: A Systematic Review and Meta-Analysis

**DOI:** 10.7759/cureus.88354

**Published:** 2025-07-20

**Authors:** Malak Maaz Hassan, Zubair Ahmad, Malak Bilal Hassan, Zoha Tasawar, Qudsia Hassan, Shafiq Ur Rahman

**Affiliations:** 1 Department of General Surgery, Hayatabad Medical Complex, Peshawar, PAK; 2 Department of Internal Medicine, Hayatabad Medical Complex, Peshawar, PAK; 3 Department of General Surgery, Saidu Teaching Hospital, Swat, PAK

**Keywords:** bariatric surgery, hypothyroidism, levothyroxine therapy, thyroid-stimulating hormone (tsh), thyroxine (t4), triiodothyronine (t3)

## Abstract

Obesity is among the most serious challenges faced by global public health. Because of the growing rate of obesity, the number of bariatric surgeries as an effective treatment is increasing day by day. Bariatric surgeries lead to significant anatomical and physiological changes in the gastrointestinal tract. Oral levothyroxine has been the treatment of choice for hypothyroidism for many years and is a narrow therapeutic index (NTI) drug, and its absorption can be significantly influenced by alterations in gastrointestinal physiology and anatomy.

The aim of this study was to evaluate the impact of bariatric surgery on levothyroxine requirements and to assess changes in thyroid function tests.

A literature search was conducted using Medical Subject Headings (MeSH) terms and relevant keywords related to surgery, bariatric procedures, hypothyroidism, and levothyroxine across PubMed, Embase, Cochrane, and ClinicalTrials.gov from inception to May 2025. Cohort studies comparing levothyroxine dose and thyroid function tests before and after bariatric surgery were included. A random-effects model using the inverse variance method was applied to account for clinical and methodological heterogeneity. Standardized mean differences (SMDs) with 95% confidence intervals (CIs) were calculated. Heterogeneity was assessed using the Chi-square (χ²) test, I² statistic, and Tau² for between-study variance. The pooled effect was tested using a Z-test, with significance set at P < 0.05.

Seventeen cohort studies with 922 patients were included in this meta-analysis. This study showed a mild and non-significant reduction in the dose of levothyroxine after bariatric surgery (SMD of 0.19 (95% CI:-0.05 to 0.42, p = 0.1) and I² = 84%) and indicated a very small and statistically non-significant improvement in favor of post-operative thyroid-stimulating hormone (TSH) (SMD is 0.03 (95% CI: -0.20 to 0.27), P=0.78, I² = 80%). There was a statistical improvement in triiodothyronine (T3) levels (SMD = 0.60; 95% CI: 0.03 to 1.17; p = 0.04 and I² = 91%) and no significant difference in T4 (SMD = 0.01; 95% CI: -0.58 to 0.60; p = 0.97; I² = 80%) levels after surgery.

This meta-analysis suggests bariatric surgery may modestly reduce levothyroxine dose and improve T3 levels in hypothyroid patients, with no significant changes in TSH or thyroxine (T4). High heterogeneity and limited study quality highlight the need for individualized dose adjustment and further robust research.

## Introduction and background

Obesity is among the most serious challenges faced by global public health. It can be defined as an abnormal or excessive build-up of body fat with a body mass index (BMI)≥30 [1]. It has many co-morbidities, including diabetes, hypothyroidism, hypertension, arthritis, and angina [2]. According to the World Health Organization (WHO), over 1 billion people worldwide are affected by obesity [1]. Because of the growing rate of obesity, the number of bariatric surgeries as an effective treatment is increasing day by day.

Bariatric surgeries such as laparoscopic sleeve gastrectomy (LSG) and gastric bypass (Roux-en-Y gastric bypass (RYGB) and single-anastomosis gastric bypass, or mini bypass) [3] lead to significant anatomical and physiological changes in the gastrointestinal tract. Sleeve gastrectomy reduces the volume of the stomach, while bypass surgeries bypass most of the stomach and small intestine [4].

Hypothyroidism is considered to be associated with morbid obesity, with a prevalence rate of 12% [5]. Oral levothyroxine has been the treatment of choice for hypothyroidism for many years and significantly helps in resolving the signs and symptoms of hypothyroidism [6]. The initial starting dose of levothyroxine is based on the patient’s weight and is then adjusted according to serum thyroid level and thyroid-stimulating hormones (TSH) [7]. However, levothyroxine is a narrow therapeutic index (NTI) drug, and its absorption can be significantly influenced by alterations in gastrointestinal physiology and anatomy [8].

Oral drug absorption is a complex, multistep process that includes disintegration, dissolution, and absorption through the intestines [9]. Each of these steps may be disrupted in bariatric surgeries. Similarly, multiple factors are involved in drug absorption from the gastrointestinal tract, including physiologic, physicochemical, and biopharmaceutical factors. A smaller stomach after the bariatric surgery with reduced gastric fluid, fewer parietal cells, impaired gastric motility, and higher gastric pH can impair drug dissolution and disintegration [10]. In bypass procedures, the upper portion of the small intestine is excluded from the digestive pathway, resulting in a reduced surface area and shorter transit time for drug absorption. Similarly, due to delayed mixing with bile and pancreatic secretions, the dissolution and absorption of lipophilic drugs are impaired. Active drug permeability may be significantly affected by bypass surgery because of the asymmetric distribution of some transporters, like P-glycoprotein and multidrug resistance-associated protein 2. Also, the expression of some metabolic enzymes like cytochrome P450 3A4 decreases aborally, resulting in higher drug blood levels after bariatric bypass surgery.

Due to these anatomic and physiological changes after bariatric surgery, the absorption of drugs and nutrients from the gastrointestinal tract may be disrupted. While malabsorption of food from the gut is desirable, changes in drug absorption may be problematic, especially for narrow therapeutic index drugs, like levothyroxine. Even a small change can result in sub-therapeutic effects or toxicity. That is why it is necessary to closely monitor thyroid function [11] and adjust levothyroxine dosing appropriately in post-bariatric surgery patients [12].

## Review

Methods and methodology

Methods

This meta-analysis was performed following the guidelines of the Cochrane Handbook for Systematic Reviews of Interventions [13]. The results are presented in accordance with the Preferred Reporting Items for Systematic Reviews and Meta-Analyses (PRISMA) statement [14]. No ethical approval was required for this study. The review protocol was registered in the International Prospective Register of Systematic Reviews (PROSPERO) under the registration ID: CRD420251065058.

Data Sources and Searches

Two independent investigators searched the Embase, MEDLINE/PubMed, Scopus, and Cochrane Library from inception until May 2025, with no date restrictions. Additionally, ClinicalTrials.gov was searched for any ongoing trials. To ensure comprehensive coverage, we also manually screened the reference lists of the included cohort studies, previously published meta-analyses, and relevant review articles.

The electronic search was conducted using a combination of Medical Subject Headings (MeSH) and the following keywords: (“Surgery, Bariatric” OR “metabolic surgery”) AND (“hypothyroidism” OR “low functioning thyroid”) AND (“levothyroxine”). There were no language restrictions. Full search strategies are given in Appendix A.

Eligibility Criteria and Outcomes

Inclusion criteria: Studies were included if they were cohort studies assessing the outcomes of bariatric surgery in hypothyroid patients taking levothyroxine. Additionally, studies had to report at least one of the selected outcomes and be published as full-text articles in peer-reviewed journals.

Exclusion criteria: Studies were excluded if they were case reports, reviews, or conference abstracts. Studies that involved subjects not taking levothyroxine before surgery were also excluded. Additionally, studies that did not report the relevant outcome data were excluded.

Outcomes: The primary outcome of this analysis was the change in the dose of levothyroxine after bariatric surgery. Secondary outcomes included changes in the values of TSH, triiodothyronine (T3), and thyroxine (T4) after surgery.

Data Extraction and Study Selection

EndNote reference management software (Clarivate, Philadelphia, PA) was used to screen all search results for duplicates. Two independent reviewers (M.B.H. and Z.A.) screened the titles and abstracts of all articles, followed by full-text assessments for the selected studies. Any discrepancies were resolved through discussion or by consulting the senior author (M.M.H.).

Data extraction was performed by two reviewers (Z.T. and Q.H.) using a standardized Microsoft Excel 2007 sheet (Microsoft Corp., Redmond, WA), and the senior author confirmed the extracted data. The extracted data included study characteristics (author, year, country, sample size, and patient demographics), intervention details (bariatric surgery), and reported outcomes.

Risk of Bias Assessment

The risk of bias and methodological quality of the included cohort studies were assessed using the Newcastle-Ottawa Scale (NOS). The studies were evaluated based on the selection of study groups, comparability of groups, and ascertainment of either the exposure or outcome of interest. A maximum of nine stars was awarded to each study, with higher scores indicating lower risk of bias and better methodological quality. Studies scoring 7-9 stars were considered high quality, 4-6 stars as moderate quality, and 0-3 stars as low quality. The assessment was performed independently by two reviewers (S.R. and Z.A.), and any discrepancies were resolved through discussion or consultation with a third reviewer (M.B.H.).

Publication Bias

To assess potential publication bias, we constructed funnel plots for outcomes, which can be found in the figures presented in Appendices B-E.

Statistical Analysis

All statistical analyses were performed using Review Manager (RevMan), version 5.4.1 (Cochrane Collaboration, London, UK), in accordance with established guidelines for conducting systematic reviews and meta-analyses. This meta-analysis was performed using the random-effects model with the inverse variance method to account for potential variability across studies due to clinical or methodological heterogeneity. Standardized mean differences (SMDs) with 95% confidence intervals (CIs) were calculated for each outcome.

Statistical heterogeneity among the included studies was evaluated using the Chi-square (χ²) test and the I² statistic. Tau² values were reported to quantify between-study variance.

An overall pooled effect was estimated using a Z-test, with significance defined at P < 0.05. Forest plots were used to graphically display individual and pooled effect sizes. Where appropriate, subgroup analyses and sensitivity analyses were performed to explore sources of heterogeneity and assess the robustness of results. Publication bias was evaluated through funnel plots.

In some studies, means and standard deviations (SDs) were not explicitly reported. So we contacted the corresponding authors via email to obtain the required data. If no response was received, we estimated the means and SDs using alternative data provided in the articles, such as interquartile ranges, medians, and sample sizes.

Sensitivity Analysis

To assess the robustness of our meta-analysis findings and determine the influence of individual studies on overall results, a leave-one-out sensitivity analysis was carried out for each outcome. In this method, one study was removed at a time, and the analysis was repeated to observe changes in the pooled effect size and heterogeneity (I²). This approach helps identify studies with disproportionate influence or methodological concerns that may affect the pooled estimates.

The primary analysis showed a small, non-significant reduction in levothyroxine dose after surgery (SMD: 0.19 (95% CI: -0.05 to 0.42), p = 0.13, I² = 84%). The exclusion of smaller or lower-quality studies reduced heterogeneity slightly but did not change the direction or statistical significance of the result. The omission of the highest-quality study (NOS 9⭐) led to increased heterogeneity and a slightly attenuated effect, suggesting its stabilizing role. The overall conclusion remained consistent: no significant change in levothyroxine requirement post-operatively.

TSH values across 12 studies showed no significant difference post-surgery (SMD: 0.03 (95% CI: -0.20 to 0.27), p = 0.78, I² = 80%). Removing some of the studies decreased I² marginally and reduced heterogeneity slightly, but did not alter the overall estimate. Across all iterations, TSH findings remained statistically nonsignificant, confirming the robustness of the result.

T4 outcomes reported in 10 studies demonstrated no statistically significant difference before and after surgery (SMD: 0.01 (95% CI: -0.58 to 0.60), p = 0.97, I² = 80%). No single study significantly influenced the results; thus, T4 outcomes appear stable and unaffected by study exclusion.

Among five studies evaluating T3 levels, a small but statistically significant improvement was observed post-operatively (SMD: 0.60 (95% CI: 0.03 to 1.17), p = 0.04, I² = 91%). Removing studies one by one did not significantly change the results.

The high heterogeneity reflects differences in follow-up durations, baseline thyroid status, and assay techniques.

Overall, the sensitivity analyses confirm that no individual study disproportionately influenced the meta-analytic findings for any of the four outcomes. While heterogeneity was high for some of the outcomes, the removal of individual studies did not meaningfully alter the conclusions. The analyses underscore that levothyroxine dose, TSH, and T4 remain statistically nonsignificant post-surgery across all scenarios. T3 shows a consistent, significant improvement, though with noted variability in magnitude among studies. These findings support the robustness of the overall results while also acknowledging the variability in study design, follow-up, and population characteristics that contribute to heterogeneity.

Results

Study Selection

**Figure 1:**
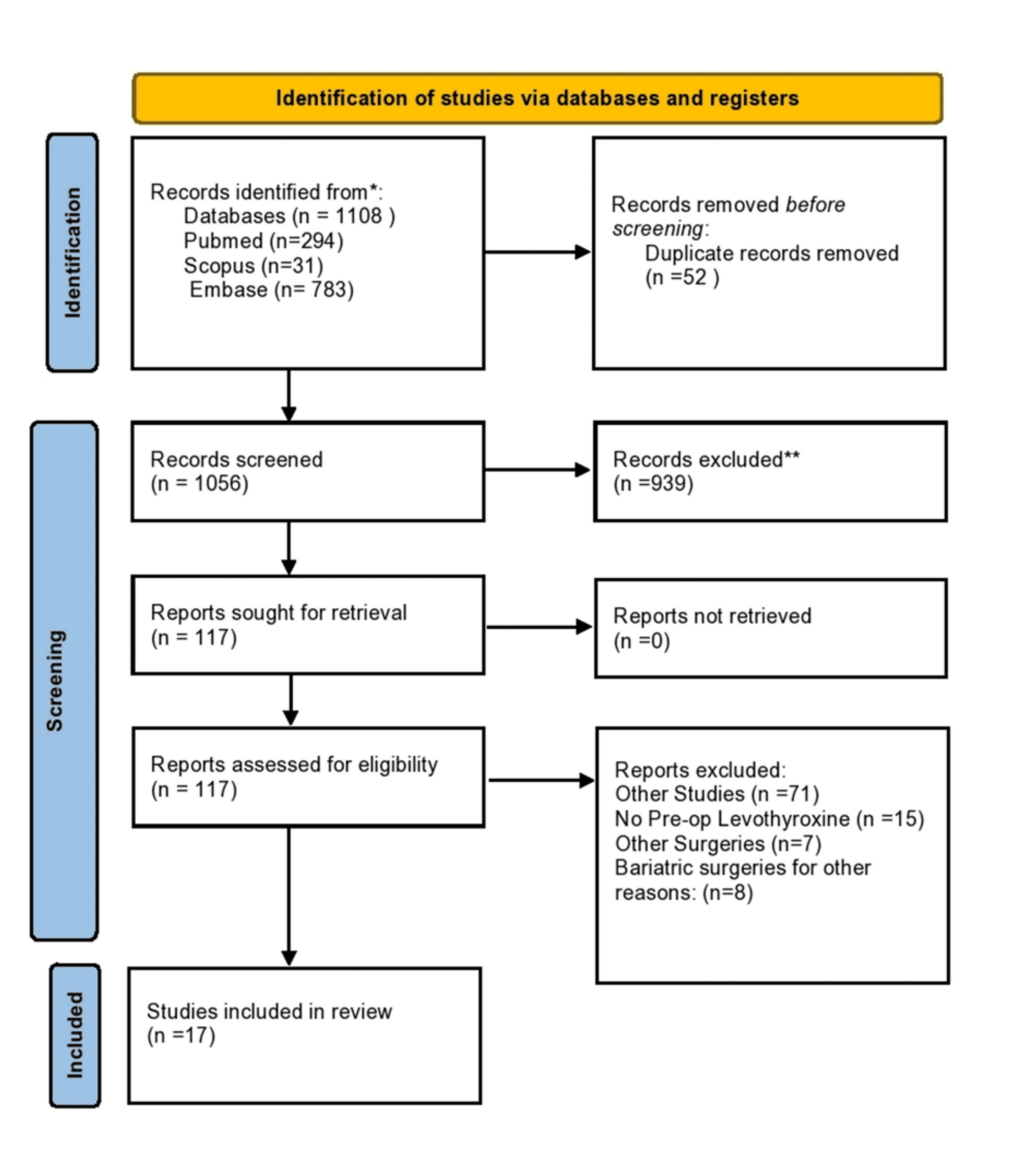
PRISMA flow chart PRISMA: Preferred Reporting Items for Systematic Reviews and Meta-Analyses

We extracted articles via electronic databases. Fifty-two duplicate articles were excluded using the EndNote (Clarivate) software and manual effort. Subsequently, 939 articles were excluded based on titles and abstracts, and 117 articles were completely retrieved and assessed. Seventeen articles (922 patients), including the inclusion and exclusion criteria, were considered in this meta-analysis (Figure 1).

Study Characteristics

The characteristics of the cohort studies are presented in Table 1. Seventeen studies with 922 patients were included in the analysis. The studies were conducted across various countries, including Saudi Arabia, Scotland, Italy, Iran, Portugal, Spain, the USA, Israel, France, Switzerland, and the Netherlands, with sample sizes ranging from 13 to 101 participants. Follow-up periods differed widely, ranging from as short as three months to up to 48 months. Participants had BMI and age ranging between 42 to 47 kg/m2 and 29.9 to 48 years, respectively. Most studies reported a higher proportion of female participants compared to male participants.

**Table 1:** Characteristics of the cohort studies

Author+year	Country	Design	Sample size	BMI	Age-median (years)	Male vs female	Follow-up (months)
Alfaifi et al. (2023) [15]	Saudi Arabia	Cohort	70	NA	NA	NA	NA
Almukainzi et al. (2024) [16]	Saudi Arabia	Cohort	101	41.62	39	10% vs 90 %	1-3, 4-6, more than 6
Dewantoro et al. (2022) [17]	Scotland	Cohort	38	49.72 ± 8.68	47.26 ± 8.89	NA	12,24,36
Fallahi et al. (2017) [18]	Italy	Cohort	13	42.9 ± 3.5	45 ± 9	4 vs 9	3-8
Fierabracchi et al. (2016) [19]	Italy	Cohort	93	45.9 ± 5.6	48 +/- 9	3 vs 90	28 ± 8
Garcia-Moreno et al. (2022) [20]	Spain	Cohort	63	44.63±6.30	48.78±10.80	5 vs 58	6,12,24
Juiz-valina et al. (2021) [21]	Spain	Cohort	48	46.01	47.2	3 vs 45	12
Julia et al. (2019) [22]	Spain	Cohort	35	43.29	45	3 vs 32	24
Malekpour et al. (2025) [23]	Iran	Cohort	60	>40	29.85 ± 7.14	24 vs 36	12
Martinelli et al. (2012) [24]	Italy	Cohort	51	45	NA	NA	NA
Patrizio et al. (2024) [25]	Italy	Cohort	69	45.22	NA	NA	3,6,12
Pedro et al. (2018) [26]	Portugal	Cohort	57	> 40 kg/m2	47	4 vs 53	12
Raftopoulos et al. (2004) [27]	USA	Cohort	23	47.5	45	3 vs 20	18
Richou et al. (2020) [28]	France	Cohort	31	42.6 ± 5.4	NA	NA	12
Trimboli et al. (2023) [29]	Switzerland	Cohort	40	42.1	48	4 vs 36	12
Yska et al. (2022) [30]	Netherland	Cohort	37	46.0 ± 7.9	43.7 ± 8.6	NA	12,24,48
Zendel et al. (2017) [31]	Israel	Cohort	93	43.7 ± 6.4	46.6 ± 11.2	8 vs 85	12

Risk of Bias Assessments

The Newcastle-Ottawa Scale (NOS) was used to assess the risk of bias across included studies. NOS evaluates the three main domains: Selection, Comparability, and Outcome assessment, with a maximum score of nine stars indicating the highest quality (Table 2).

**Table 2:** Risk of bias of the included studies Each ⭐ represents one point awarded based on the Newcastle-Ottawa Scale (NOS) criteria, evaluating three domains: selection, comparability, and outcome/exposure. The maximum score is 9 stars, indicating the highest methodological quality.

Study	Selection				Comparability	Outcomes			Total
	Representativeness of exposed cohort	Selection of non-exposed cohort	Ascertainment of exposure	Outcome not present at the start of the study		Assessment of outcomes	Length of follow-up	Adequacy of follow-up	
Alfaifi et al. (2023) [15]	⭐	-	⭐	⭐	-	⭐	⭐	⭐	6⭐
Almukainzi et al. (2024) [16]	⭐	⭐	⭐	⭐	⭐	⭐	⭐	-	7⭐
Dewantoro et al. (2022) [17]	⭐	⭐	⭐	⭐	⭐	⭐	⭐	-	7⭐
Fallahi et al. (2017) [18]	⭐	-	⭐	⭐	-	⭐	⭐	⭐	6⭐
Fierabracchi et al. (2016) [19]	⭐	-	⭐	⭐	⭐	⭐	⭐	⭐	7⭐
Garcia-Moreno et al. (2022) [20]	⭐	-	⭐	⭐	⭐	⭐	⭐	⭐	7⭐
Juiz-valina et al. (2021) [21]	⭐	-	⭐	⭐	⭐	⭐	⭐	⭐	7⭐
Julia et al. (2019) [22]	⭐	-	⭐	⭐	⭐	⭐	⭐	⭐	7⭐
Malekpour et al. (2025) [23]	⭐	-	⭐	⭐	-	⭐	⭐	⭐	6⭐
Martinelli et al. (2012) [24]	⭐	-	⭐	⭐	-	⭐	-	-	4⭐
Patrizio et al. (2024) [25]	⭐	⭐	⭐	⭐	⭐	⭐	⭐	-	7⭐
Pedro et al. (2018) [26]	⭐	⭐	⭐	⭐	⭐	⭐	⭐	-	7⭐
Raftopoulos et al. (2004) [27]	⭐	-	⭐	⭐	-	⭐	⭐	-	5⭐
Richou et al. (2020) [28]	⭐	-	⭐	⭐	-	⭐	⭐	-	5⭐
Trimboli et al. (2023) [29]	⭐	-	⭐	⭐	-	⭐	⭐	-	5⭐
Yska et al. (2022) [30]	⭐	⭐	⭐	⭐	⭐⭐	⭐	⭐	⭐	9⭐
Zendel et al. (2017) [31]	⭐	-	⭐	⭐	-	⭐	⭐	⭐	6⭐

Most studies have a strong methodology in the selection and outcome assessment domains, but have a key limitation in the comparability of cohorts. Many of the included studies scored between 6 to 7 stars and were considered moderate to good quality. The study by Yska et al. (2022) scored 9 stars and achieved a high quality [30]. On the contrary, the study by Martinelli (2012) was rated low quality with a score of only 4 stars, mainly due to a lack of comparability and incomplete data [24]. Similarly, the studies by Alfaifi et al. (2023) and Fallahi et al. (2017) were low-scoring due to a possible risk of bias due to uncontrolled confounding variables [15,18].

Overall, the risk of bias among the studies was considered low to moderate, and these variations were accounted for in sensitivity analyses to evaluate their impact on the meta-analysis findings.

Primary Outcome

**Figure 2:**
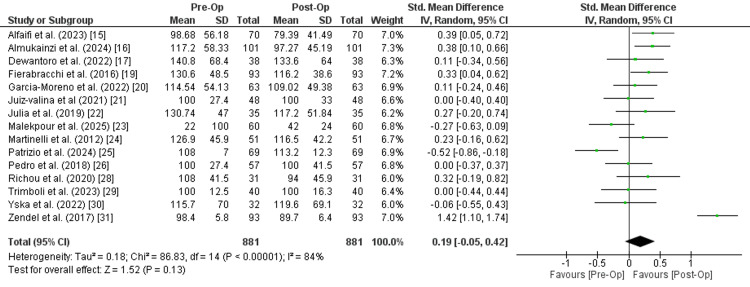
Meta-analysis of levothyroxine dose

Levothyroxine dose: Fifteen studies with a combined sample size of 881 participants compared levothyroxine doses before and after surgery. The reported data indicated a small, non-significant effect favoring post-operative low-dose requirement of levothyroxine with an overall SMD of 0.19 (95% CI:-0.05 to 0.42). The test for overall effect yields a Z-value of 1.52 (p = 0.13), suggesting that the observed difference is not statistically significant. Also, there is significant heterogeneity among the studies with an I² of 84% and a Chi² of 86.83 (df = 14, p < 0.00001), showing a substantial variability in the effect sizes across studies. Most of the studies favor post-operative findings, but some studies show little to no effect. This variation in the studies may be due to differences in the study population, interventions, or outcome measures (Figure 2).

Secondary Outcomes

**Figure 3:**
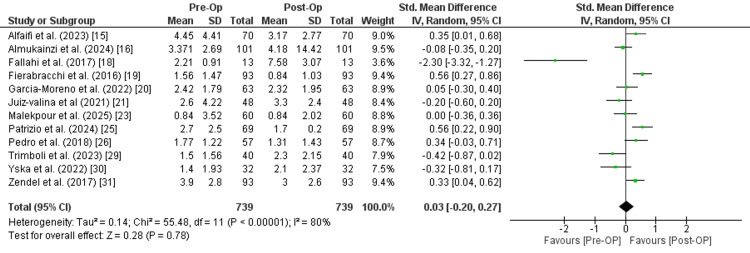
Meta-analysis of thyroid-stimulating hormone

TSH: Twelve studies with a combined sample size of 739 patients reported the value of TSH before and after surgery. The reported data indicated a very small and statistically non-significant effect in favor of post-operative outcome (SMD is 0.03 (95% CI: -0.20 to 0.27), Z=0.28, P=0.78). The confidence interval crosses zero, suggesting no clear or consistent difference between pre-operative and post-operative values across the included studies. Moreover, there is significant heterogeneity among the studies (I² = 80%, Chi² = 55.48, p < 0.00001), indicating variability in study populations, interventions, or outcome measures (Figure 3).

**Figure 4:**
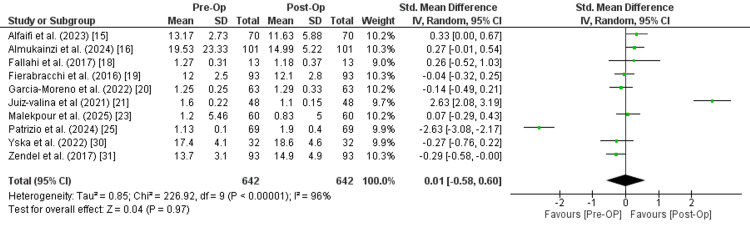
Meta-analysis of thyroxine (T4)

T4: Ten studies with a combined sample size of 642 patients reported the value of T4 before and after surgery. The reported data indicated no statistically significant difference between the pre-operative and post-operative measurements (SMD is 0.01 (95% CI: -0.58 to 0.60), Z=0.04, P=0.97). The included studies demonstrated a significant heterogeneity (I² = 80%, Chi² = 55.48, p < 0.00001), suggesting substantial variability in study outcomes (Figure 4).

**Figure 5:**
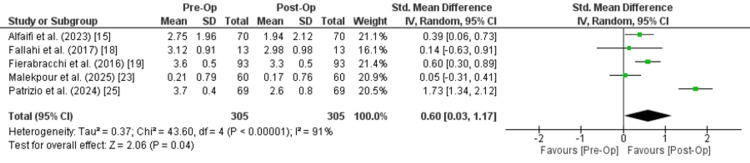
Meta-analysis of triiodothyronine (T3)

T3: Five studies, including 305 patients, assessed the value of T3 before and after surgery. The analysis indicated a statistically significant improvement after the surgery with an SMD of 0.60 (95% CI: 0.03 to 1.17; P=0.04), favoring post-operative values. However, considerable heterogeneity was observed across the studies (I² = 91%, Chi² = 43.60, df = 4, P < 0.00001), indicating notable variation in study results. Despite this heterogeneity, the overall analysis suggests a statistically significant benefit after the surgery (Figure 5).

Certainty of Evidence

The certainty of evidence for each outcome was assessed using the GRADE (Grading of Recommendations, Assessment, Development, and Evaluation) approach. Two of the outcomes were rated as low and two as moderate certainty, mainly due to heterogeneity and potential risk of bias. Table 3 summarizes the main findings and certainty of evidence for each outcome.

**Table 3:** Certainty of evidence for each outcome Certainty of evidence was assessed using the GRADE (Grading of Recommendations, Assessment, Development, and Evaluation) approach. Each filled circle (⬤) indicates one level of confidence in the estimate of effect, with four circles (⬤⬤⬤⬤) representing high certainty and fewer circles indicating lower certainty. SMD: standardized mean difference; TSH: thyroid-stimulating hormone; T4: thyroxine; T3: triiodothyronine

Outcome	Relative effect (95% CI)	No. of participants (Studies)	Certainty of evidence	Comments
Levothyroxine dose	SMD = 0.19 (−0.05 to 0.42)	881 (15 studies)	⬤⬤⬤◯ Moderate	Downgraded for inconsistency due to high heterogeneity (I² = 84%). Effect not statistically significant.
TSH	SMD = 0.03 (−0.20 to 0.27)	739 (12 studies)	⬤⬤◯◯ Low	Downgraded for imprecision and inconsistency (I² = 80%). Null effect; wide confidence interval.
T4	SMD = 0.01 (−0.58 to 0.60)	642 (10 studies)	⬤⬤◯◯ Low	Downgraded for very high heterogeneity (I² = 96%) and imprecision. Wide CI crossing both benefit and harm.
T3	SMD = 0.60 (0.03 to 1.17)	305 (5 studies)	⬤⬤⬤◯ Moderate	Downgraded for inconsistency (I² = 91%). Effect is statistically significant, but heterogeneity is substantial.

Discussion

Our meta-analysis of 17 studies involving 922 patients shows a statistically significant improvement in T3 levels after surgery. There was a slight reduction in levothyroxine dose and an improvement in TSH levels; however, neither was statistically significant. No significant change was observed in T4 levels.

Our meta-analysis shows that the levothyroxine requirement reduces slightly after bariatric surgery. These findings are supported by previous studies. For example, a meta-analysis by Azran et al. (2021) analyzed 28 studies involving 1,284 patients who underwent bariatric surgery and found a statistically significant reduction in levothyroxine dose postoperatively (mean difference = −13.20 mcg/day, 95% CI: −19.69 to −6.71) [32]. Similarly, a systematic review by Gadiraju et al. (2016), including 10 studies, also reported reduced levothyroxine requirements following surgery [33]. Consistent results were reported by Barzin et al. (2024) [34]. Collectively, these studies support our finding of reduced levothyroxine requirement after bariatric surgery.

In contrast, some studies have reported increased levothyroxine requirements postoperatively. For instance, a study by Martirosyan & Danielyan (2024) found higher dose requirements in certain patients [35]. Additionally, Barzin et al. (2024) noted that by the third year of follow-up, while 56.1% of sleeve gastrectomy patients and 33.3% of one-anastomosis gastric bypass patients experienced a reduction in levothyroxine dose, 24.4% and 9.1%, respectively, required dose increases [34].

The decrease in levothyroxine requirements after bariatric surgery can be related to weight loss or other physiological changes. The loss of both lean body mass and fat significantly reduces the overall metabolic need for thyroxine. Additionally, improvements in the changed pharmacokinetics of levothyroxine seen in obesity (such as enhanced absorption, distribution, and metabolism) likely contribute to the postoperative reduction in hormone dosage. Moreover, the reversal of obesity-related changes, such as impaired hormone metabolism, systemic inflammation, and a high set point of thyroid hormone homeostasis, likely contributed to the postoperative decrease in levothyroxine needs. The degree of dose reduction may vary depending on the type of surgery. For instance, sleeve gastrectomy primarily influences levothyroxine requirements through weight loss, whereas gastric bypass may also involve altered drug absorption due to changes in the anatomy of the gastrointestinal system.

Clinical adjustments in levothyroxine dosing are typically based on thyroid function tests, the type of surgery performed, and shifts from total to ideal body weight for more accurate hormone replacement.

Regarding postoperative TSH levels, our meta-analysis demonstrated a slight, though statistically nonsignificant, decrease following bariatric surgery. This trend is supported by previous individual studies. Tian et al. (2024) reported a significant reduction in serum TSH levels, from 2.33 (1.67-3.04) uIU/mL preoperatively to 1.82 (1.21-2.50) uIU/mL postoperatively (P < 0.001) [36]. Similarly, Granzotto et al. (2020), in a retrospective review of 215 patients, found that weight loss after bariatric surgery led to normalization of TSH levels in most patients, with none developing overt hypothyroidism [37]. Khazraei et al. (2024) also observed a significant decrease in TSH levels six months after surgery (P = 0.005) [38]. Obesity is usually associated with low-grade chronic inflammation and altered hypothalamic-pituitary-thyroid axis regulation, resulting in deranged TSH. Weight loss, improved thyroid hormone sensitivity, and changes in peripheral metabolism after bariatric surgery help in improving the TSH level 

Similarly, this meta-analysis showed statistical improvement of T3 but no improvement in the value of T4 after surgery. Many previous studies showed the same findings, such as Tian et al. (2024) and Khazraei et al. (2024), supporting our findings [36,38].

Moreover, with the GRADE approach, we found that the certainty of evidence for levothyroxine dose and T3 was moderate, while most of that of TSH and T4 was rated as low. This is attributed to imprecision, heterogeneity, and the subjective nature of outcome assessment. These limitations underscore the need for more standardized and methodologically rigorous trials in the future.

Our meta-analysis has some limitations as well. First, inconsistency in the timing of follow-ups may have affected outcomes. Second, all the studies included in our meta-analysis were cohorts. Third, variability in patient characteristics, such as age, comorbidities, and demographic factors, may have contributed to differences in outcomes.

## Conclusions

This meta-analysis suggests that bariatric surgery in hypothyroid patients on levothyroxine may lead to a modest reduction in levothyroxine dose and a statistically significant improvement in serum T3 levels, though changes in TSH and T4 were not statistically significant. Due to high heterogeneity, variability in baseline characteristics, and the observational nature of the included studies, close postoperative monitoring and individualized dose adjustment remain essential. Given the moderate to low certainty of evidence and high heterogeneity, further high-quality research is needed to confirm these findings.

## Figures and Tables

**Figure 6:**
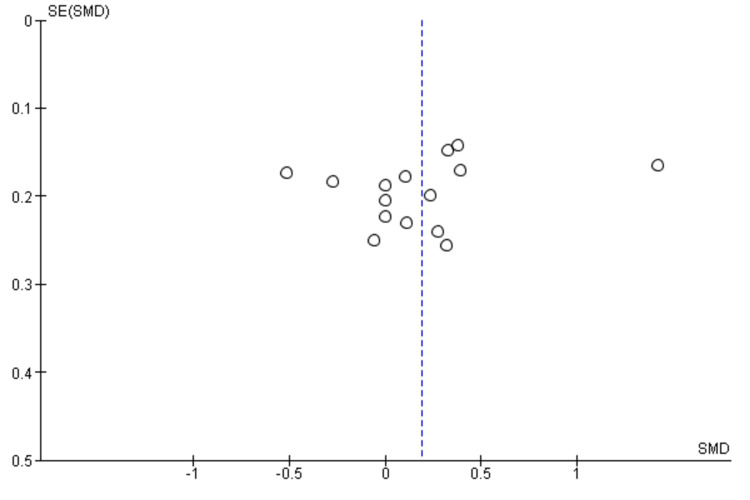
Levothyroxine funnel plot of the included studies SE(SMD): Standard Error of the Standardized Mean Difference

**Figure 7:**
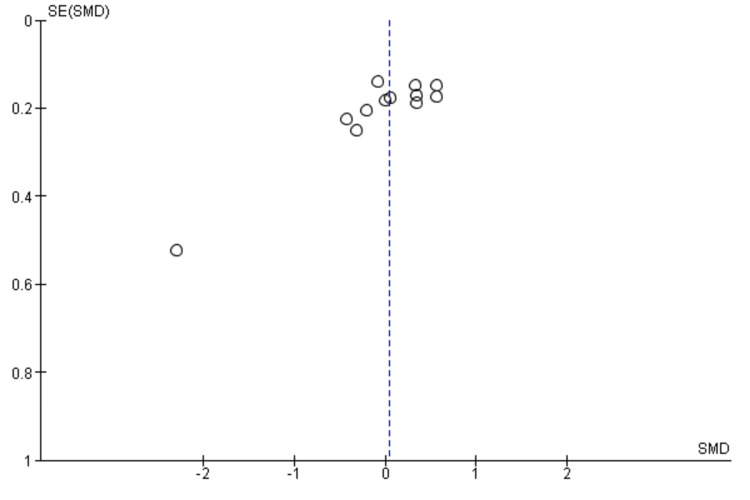
TSH funnel plot of the included studies SE(SMD): Standard Error of the Standardized Mean Difference; TSH: thyroid-stimulating hormone

**Figure 8:**
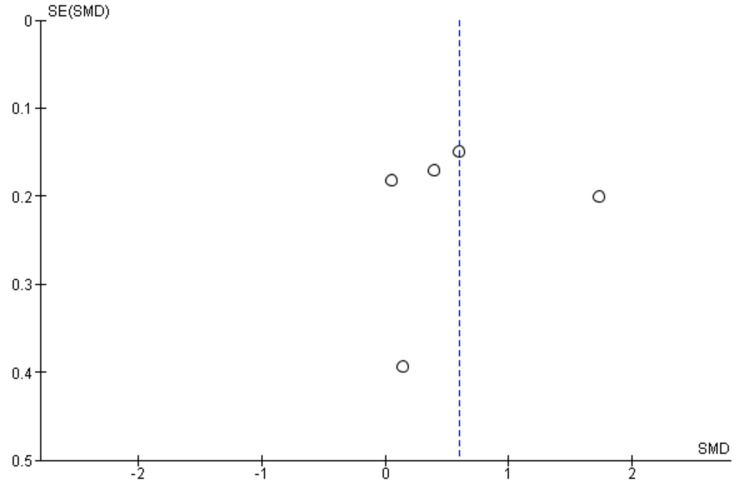
T3 funnel plot of the included studies SE(SMD): Standard Error of the Standardized Mean Difference; T3: triiodothyronine

**Figure 9:**
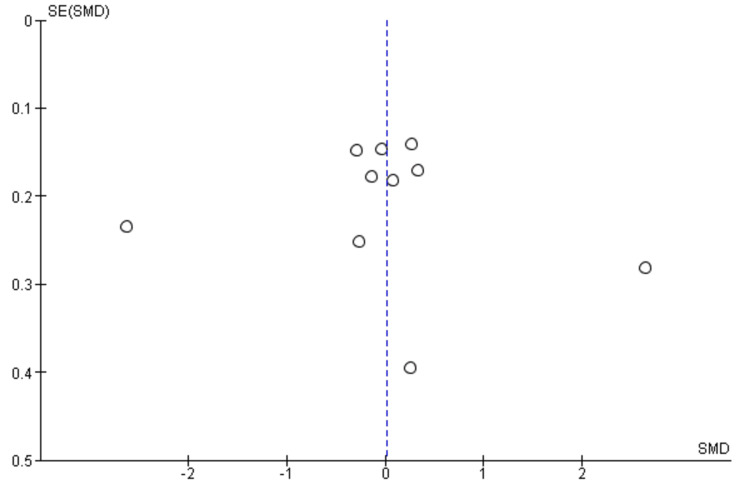
T4 funnel plot of the included studies SE(SMD): Standard Error of the Standardized Mean Difference; T4: thyroxine

## Appendices

Appendix A 

**Table 4:** Search strategy; population and intervention with their MeSH terms MeSH: Medical Subject Headings

Population	Intervention	Population
Hypothyroidism	Bariatric surgery	Levothyroxine
"hypothyroidism"[MeSH Terms]	"bariatric surgery"[MeSH Terms] Surgeries, Bariatric Surgery, Bariatric Surgeries, Metabolic Surgery, Metabolic Surgeries, Metabolic Bariatric Surgical Procedure, Bariatric Surgical Procedures, Bariatric Surgical Procedure, Bariatric Stomach Stapling, Stomach	Levothyroxine

Appendix B 

Appendix C 

Appendix D 

Appendix E 
